# Mining metagenomic data to gain a new insight into the gut microbial biosynthetic potential in placental mammals

**DOI:** 10.1128/spectrum.00864-24

**Published:** 2024-08-20

**Authors:** Dini Hu, Tongzuo Zhang, Shunfu He, Tianchun Pu, Yanqiang Yin, Yibo Hu

**Affiliations:** 1 Key Laboratory of Animal Ecology and Conservation Biology, Institute of Zoology, Chinese Academy of Sciences, Beijing, China; 2 Key Laboratory of Adaptation and Evolution of Plateau Biota, Northwest Institute of Plateau Biology, Chinese Academy of Sciences, Xining, Qinghai, China; 3 Xining Wildlife Zoo, Xining, Qinghai, China; 4 Beijing Zoo, Beijing, China; 5 Chongqing Zoo, Chongqing, China; Chengdu University, Chengdu, Sichuan, China

**Keywords:** metagenomics, gut microbiome, mammals, biosynthesis

## Abstract

Mammals host a remarkable diversity and abundance of gut microbes. Biosynthetic gene clusters (BGCs) in microbial genomes encode biologically active chemical products and play an important role in microbe–host interactions. Traditionally, the exploration of gut microbial metabolic functions has relied on the pure culture method. However, given the limited amounts of microbes being cultivated, insights into the metabolism of gut microbes in mammals continued to be very limited. In this study, we adopted a computational pipeline for mining the metagenomic data (named taxonomy-guided identification of biosynthetic gene clusters, TaxiBGC) to identify experimentally verified BGCs in 373 metagenomes across 53 mammalian species in an unbiased manner. We demonstrated that polyketides (PKs) and nonribosomal peptides (NRPs) are representative of mammals, and the products derived from them were associated with cell–cell communication and resistance to inflammation. Large carnivores had the highest number of BGCs, followed by large herbivores and small mammals. We also observed that the large mammals had more common BGCs that aid in the biosynthesis of a variety of natural products. However, small mammals not only had fewer BGCs but were also unique to each species. Our results provide novel insights into the mining of metagenomic data sets to identify active BGCs and their products across mammals.

**IMPORTANCE:**

The gut microbes host numerous biosynthetic gene clusters (BGCs) that biosynthesize natural products and impact the host’s physiology. Historically, our understanding of BGCs in mammalian gut microbes was largely based on studies on cultured isolates; however, only a small fraction of mammal-associated microbes have been investigated. The biochemical diversity of the mammalian gut microbiota is poorly understood. Metagenomic sequencing contains data from a vast number of organisms and provides information on the total gene content of communities. Unfortunately, the existing BGC prediction tools are designed for individual microbial genomes. Recently, a BGC prediction tool called the taxonomy-guided identification of biosynthetic gene clusters (TaxiBGC) that directly mine the metagenome was developed. To gain new insights into the microbial metabolism, we used TaxiBGC to predict BGCs from 373 metagenomes across 53 mammalian species representing seven orders. Our findings elucidate the functional activities of complex microbial communities in the gut.

## INTRODUCTION

Mammals require adequate nutrition and sizeable food from animal or plant sources. Diverse habitats and the complexity of mammalian digestive systems promote the formation of a remarkable abundance of microbes. These microbes develop certain symbiotic relationships with their hosts, depending on the molecular interactions between the symbiotic partners (1). There is increasing evidence that microbes have huge potential to biosynthesize a wide range of small molecular compounds with bioactivities (2). These metabolites have promising therapeutic function, and the ability to regulate host–microbe and microbe–microbe interactions (3), such as the modulation of immune and pro-inflammatory responses, and function as a barrier to prevent pathogen invasion (4–6).

Exploring the mechanisms of interactions between gut microbes and their hosts has led to increased interest in the corresponding secondary metabolites. Gut microbes produce natural products in two distinct ways (7). First, they produce metabolites from dietary components, such as tryptophan, short-chain fatty acids, and oligosaccharides. Second, they produce uncharacterized end products from unique biosynthetic gene clusters (BGCs). Over the past few decades, numerous BGC-derived natural products were discovered in the mammalian gut, and these distinct compounds play important roles in mediating significant interactions and physiological functions for host survival. The BGCs contain genes associated with immunity and transport. Consequently, BGC-derived natural products from the mammalian gut microbiota have an impact on the physiology of an individual (8). Most of these peptides were characterized as ribosomally synthesized and post-translationally modified peptides (RiPPs). Nisin H produced by *Streptococcus hyointestinalis* DPC6484 inhibits a wide range of Gram-positive bacteria (9). Ruminococcin C from the gut microbe *Ruminococcus gnavus* E1 exhibits activity against pathogens (10). Clostridiolysin S is responsible for a hemolytic phenotype (11). However, polyketides (PKs) and non-ribosomal peptides (NRPs), which are key precursors of numerous therapeutic molecules, are derived from the mammalian gut microbiota (8, 12, 13). The expanded chemical capabilities of gut microbes allow us to understand host–microbe interactions.

Pure culture has long been the traditional approach toward discovering and identifying BGC-derived natural products (14, 15). After isolating specific bacteria, BGCs and their products can be identified and purified using genome mining and chemical analyses. Historically, our understanding of the metabolism of mammalian gut microbes was largely based on studies on cultured isolates, whereas only a small fraction of mammal-associated microbial metabolic pathways, enzymes, and metabolites have been investigated *in vivo*. The biochemical diversity of the mammalian gut microbiota is poorly understood (3). Identifying the genes responsible for the gut microbial metabolic and biosynthetic capabilities is crucial to understanding the contribution of these organisms to host health. Owing to the advancements in molecular biology and development of new technologies for metagenomic analysis, the metabolism of mammalian gut microbes in their natural habitats can be studied. Metagenomic sequencing contains data from a vast number of organisms and provides information on the total gene content of communities, including taxonomic and functional genes. Traditional BGC prediction tools such as antiSMASH (16), CLUSEAN (17), and PRISM (18) are designed for individual microbial genomes. Direct characterization of BGCs from metagenomic reads is usually based on biosynthetic SPAdes (19) and MetaBGC (20). However, both of the currently widely used metagenome-based BGC prediction approaches are limited by the requirement of read assembly, considerable computational time, and unknown microbes containing the BGCs. Recently, an easy-to-use, rapid, and unbiased BGC prediction tool called the taxonomy-guided identification of biosynthetic gene clusters (TaxiBGC) was developed (21). TaxiBGC does not require genome assembly and can be applied to any metagenome. This novel approach facilitates the prediction of experimentally characterized BGCs by considering the source microbial species, thereby enabling users to trace their predicted taxonomic origins.

Exploring the biochemical functions of mammalian metagenomes and elucidating their effects on host biology are critical. In this study, we analyzed the gut microbial communities of various mammalian species across habitats, diets, body sizes, and threatened classes to understand the contribution of microbes to the biosynthesis of natural products that benefit the host. We used previously published metagenomic data generated from metagenomic sequencing of fecal DNA from 37 mammalian species. We also collected 54 fecal samples from 16 *Felidae* species that had undergone metagenomic sequencing. First, the BGCs and associated products of these species were predicted using the TaxiBGC pipeline. We compared the composition of BGCs across mammalian orders and the relative contribution of different orders to BGC biosynthesis, given the differences in interactions between gut microbes and the host. Furthermore, we identified critical parameters that differentiate the composition of BGCs among mammals. Overall, our study offers a large-scale and comprehensive assessment of the biosynthetic potential of gut microbiota in mammals based on metagenomes. These results provide a complementary overview of encoded mammalian microbial functionalities and elucidate host–microbe interactions. Furthermore, the metabolic products of these microorganisms provide a vast opportunity for chemists because natural products with biological activities are directly derived from BGCs. The discovery and identification of biosynthetic genes can accelerate the prediction of structural and functional properties of metabolites that are crucial to initiating production and guide isolation strategies.

## RESULTS

### Experimentally characterized BGCs in the mammalian metagenome

**Fig 1:**
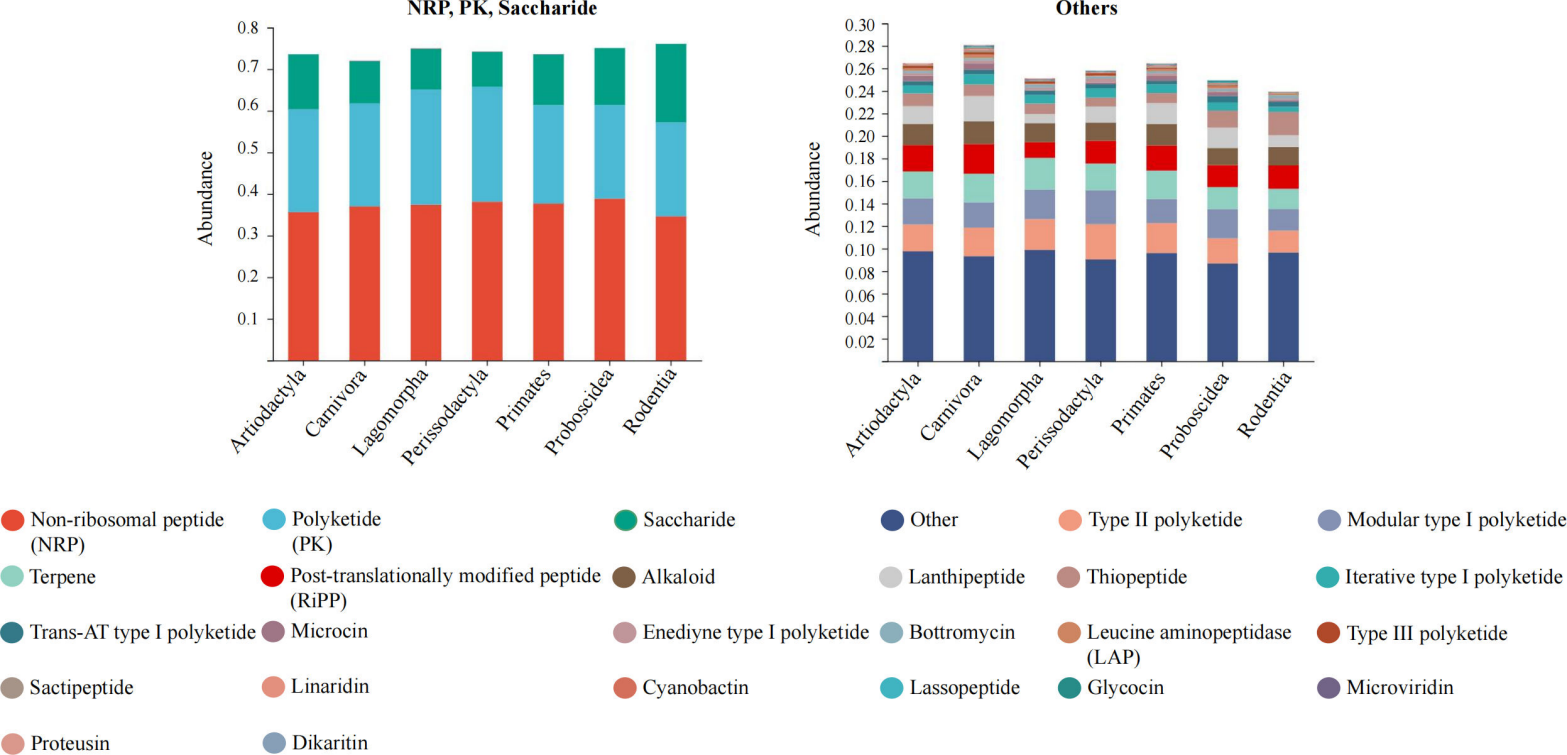
Number of BGCs in each mammalian order.

**Fig 2:**
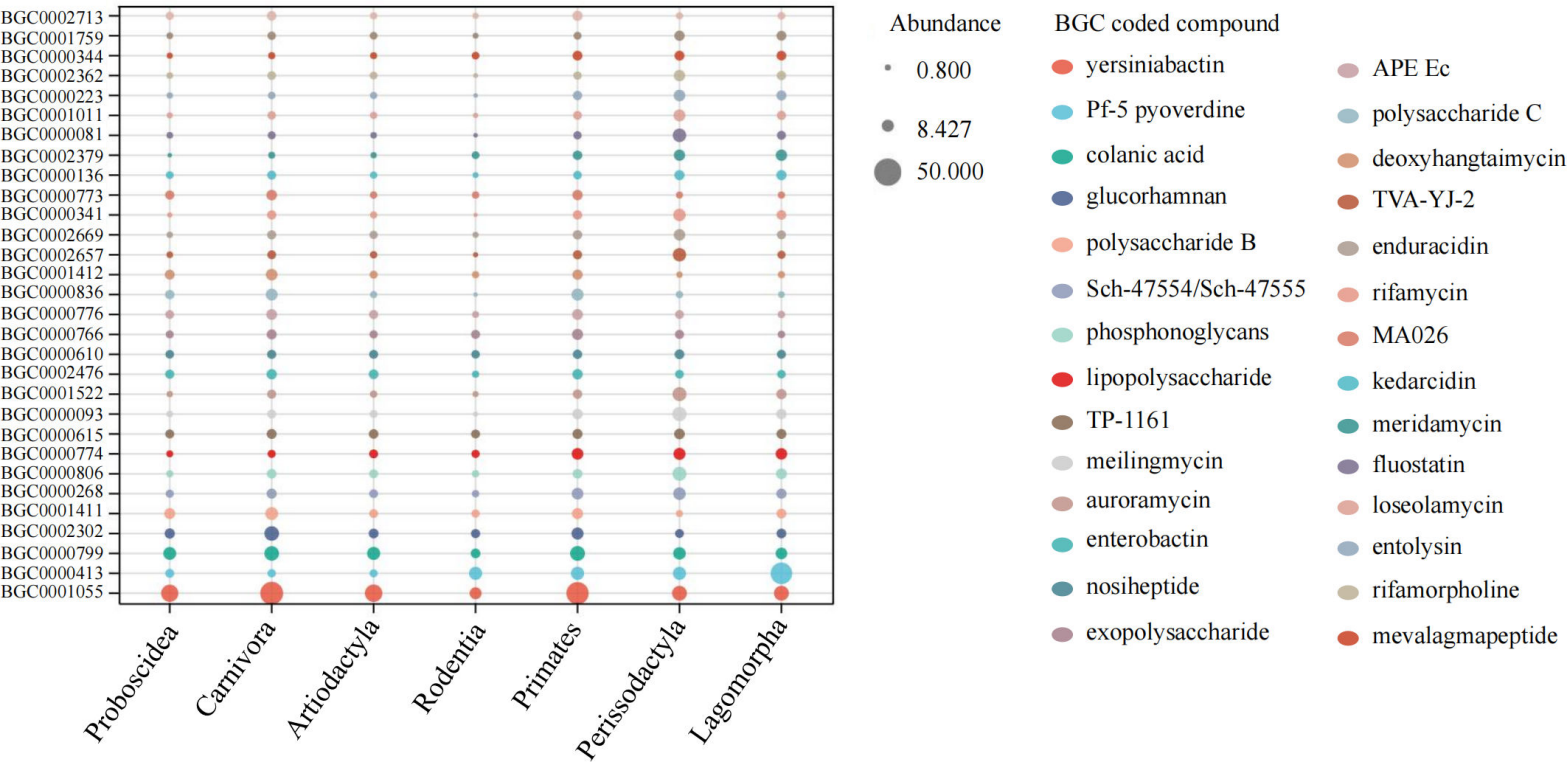
Biosynthetic gene clusters ranked according to the number in each mammalian order. The larger bubble represents the higher number of BGCs.

**Fig 3:**
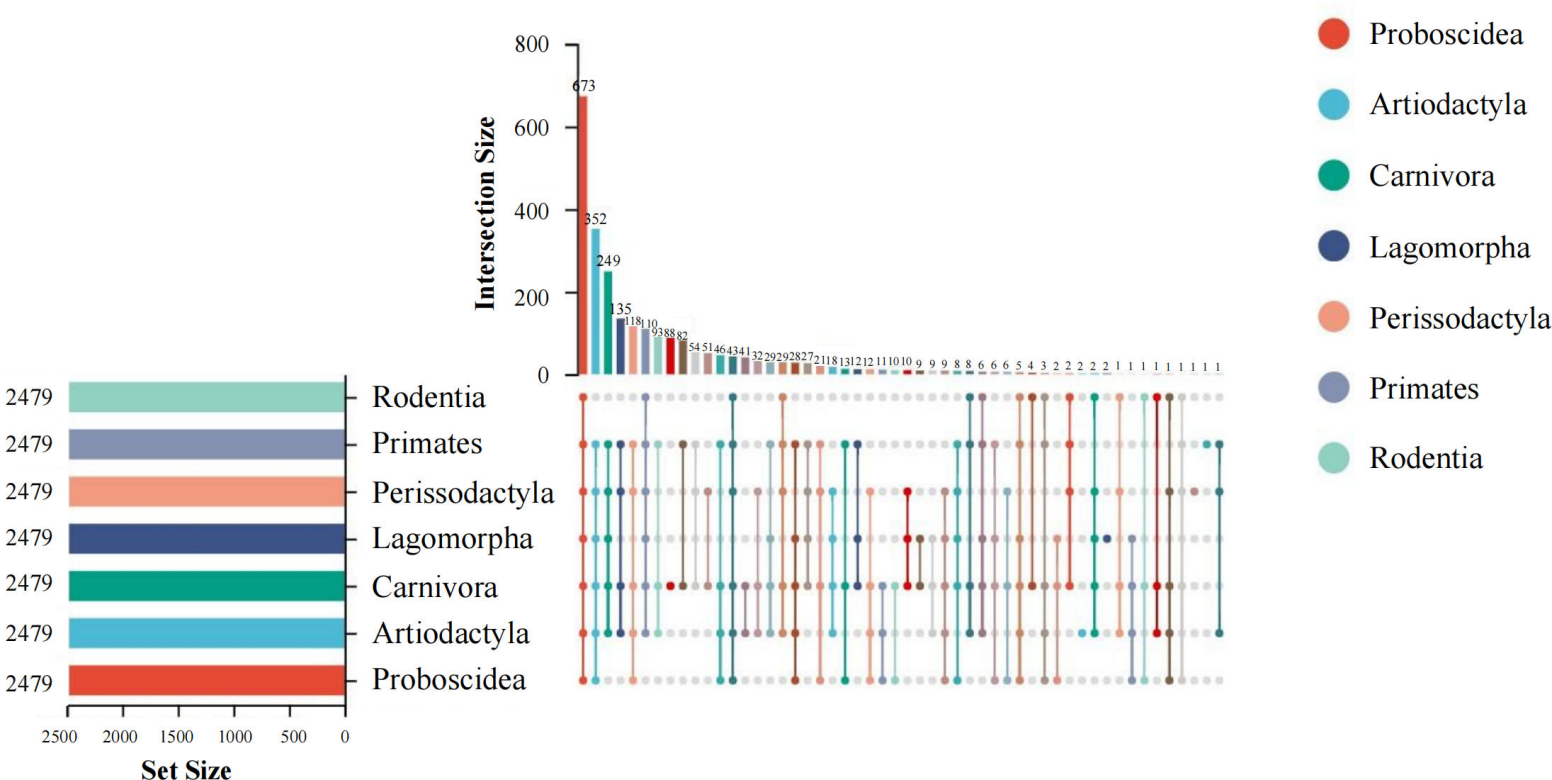
UpSet plot showing the number of shared and unique BGCs among seven orders.

We generated a mammalian gut metagenome from a variety of diverse species spanning seven orders (Artiodactyla, Carnivora, Lagomorpha, Perissodactyla, Primates, Proboscidea, and Rodentia) and 18 families (Table S1). In total, 373 samples passed quality control. Fifty-three mammal species covering a wide range of diets, body sizes, habitats, and threatened classes were represented, with up to 48 individuals per species (mean of 5). The presence of BGCs was determined using the TaxiBGC pipeline. The TaxiBGC database includes experimentally characterized BGCs and their corresponding secondary metabolites, along with their source organisms (microbial species and/or strains). We identified 2,479 unique experimentally characterized BGCs that were classified into 25 biosynthetic classes in at least one sample (Table S2). Biosynthetic gene clusters encoding NRPs were the most prevalent, followed by PKs and saccharides (Fig. 1). The most abundant BGCs were found in Carnivora (2,472), followed by Primates (2,155), Artiodactyla (2,068), Perissodactyla (1,966), Lagomorpha (1,567), Proboscidea (1,370), and Rodentia (890). The dominant BGCs were BGC000105 (yersiniabactin), BGC0000413 (Pf-5 pyoverdine), BGC0000799 (colanic acid), BGC0002302 (glucorhamnan), and BGC0001411 (polysaccharide B) (Fig. 2). Of these, 673 BGCs were shared among all seven orders, and 88 BGCs only appeared in Carnivora (Fig. 3). The remaining six orders had no distinct BGCs (Fig. 3). The four most common BGCs were yersiniabactin, Pf-5 pyoverdine, colanic acid, and glucorhamnan. All BGCs present only in Carnivora showed a relatively low abundance (mean value of 1).

### Differences and similarities of BGCs across mammalian orders

**Fig 4:**
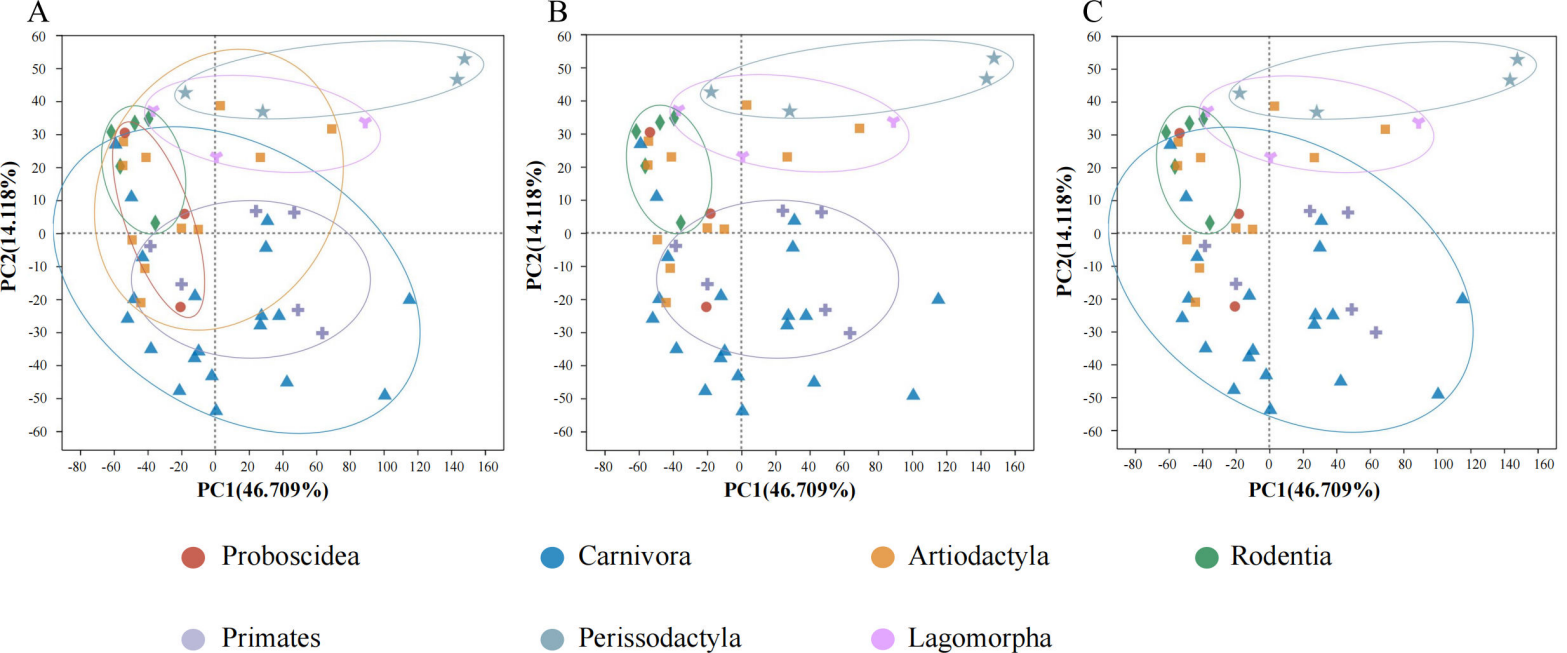
Principal component analysis plot showing the similarities or differences in the composition of BGCs among seven orders. The individuals from the same order were clustered together (**A**). For the different orders, Perissodactyla, Lagomorpha, Rodentia, and Primates were separated, (**B**) and Perissodactyla, Lagomorpha, Rodentia, and Carnivora were separated (**C**).

**Fig 5:**
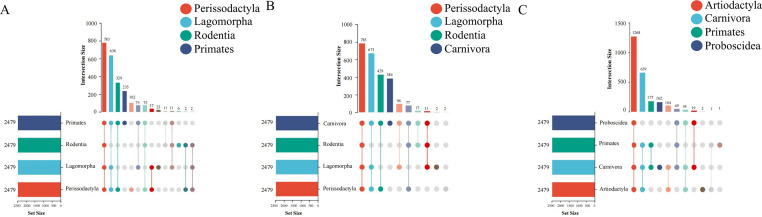
UpSet plot showing the number of shared and unique BGCs among Perissodactyla, Lagomorpha, Rodentia, and Primates. (**A**) Perissodactyla, Lagomorpha, Rodentia, and Carnivora (**B**) and Proboscidea, Carnivora, Artiodactyla, and Primates (**C**).

Subsequently, we evaluated the contribution of host phylogeny to the composition of BGCs and found that samples from the same order could be clustered in the principal component analysis (PCA) graph, whereas samples from different orders could not be completely separated (Fig. 4A, ANOSIM, *P* = 0.013). These differences were first reflected in the separation of Perissodactyla, Lagomorpha, Rodentia, and Primates (Fig. 4B, ANOSIM, *P* = 0.008). The gut metagenome of Primates was associated with the production of colicin V (BGC0000584), N-acyloxyacyl glutamine (BGC0001684), tryglysin B (BGC0002583), thiocillin I (BGC0000612), and lacticin Q (BGC0000620) (Fig. 5A). The four BGCs (encoding kocurin, lagmysin, tacrolimus, and cacibiocin B) were abundant and only present in Perissodactyla (Fig. 5A). Geosmin (BGC0000661) and pyrrolnitrin (BGC0000924) were only found in the guts of Lagomorpha (Fig. 5A). All BGCs associated with Rodentia were low in abundance. Furthermore, the composition of BGCs could be differentiated among Perissodactyla, Lagomorpha, Rodentia, and Carnivora (Fig. 4C, ANOSIM, *P* = 0.004). Carnivora may encode for 12 BGCs, namely, colicin V (BGC0000584), microcin C7 (BGC0000585), lacticin Q (BGC0000620), microcin J25 (BGC0000581), clostridiolysin S (BGC0000564), thiocillin I (BGC0000612), gallidermin (BGC0000514), clostridiolysin S (BGC0001170), carnocyclin (BGC0000487), capsular polysaccharide (BGC0000731), aranazole (BGC0001884), and aerobactin (BGC0001498) (Fig. 5B). The similarities in BGCs among Proboscidea, Carnivora, Artiodactyla, and Primates were determined (Fig. 4A). The top shared BGCs were yersiniabactin (BGC0001055), Pf-5 pyoverdine (BGC0000413), glucorhamnan (BGC0002302), colanic acid (BGC0000799), polysaccharide (BBGC0001411), meilingmycin (BGC0000093), polysaccharide (BGC0000792), APE Ec (BGC0000836), MA026 (BGC0002379), Sch-47554 and Sch-47555 (BGC0000268), polysaccharide C (BGC0001412), phosphonoglycans (BGC0000806), and deoxyhangtaimycin (BGC0002657) (Fig. 5C). We found that host phylogeny cannot be used as a factor to distinguish the composition of mammalian BGCs but can only separate several orders.

### Variation of BGCs in mammals caused by habitat, diet, body size, and threatened classes

**Fig 6:**
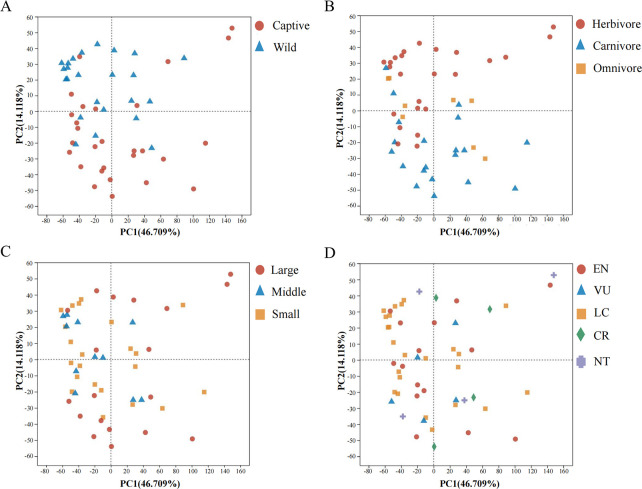
Principal component analysis plot showing the similarities or differences in the composition of BGCs between wild and captive samples (**A**) among herbivores, carnivores, and omnivores (**B**); among large, middle, and small body size (**C**); and among threatened classes [D, endangered (EN), vulnerable (VU), least concern (LC), critically endangered (CR), and near threatened (NT)].

Furthermore, we determined the effects of habitat, diet, body size, and threatened classes (threatened: CR, EN, and VU; non-threatened: LC and NT) on BGC biosynthesis. The analysis of molecular variance (AMOVA) analysis was used to explore factors that significantly changed the composition of BGCs in mammals. The composition of BGCs in wild mammals differed significantly from that in captive mammals (AMOVA, *P* = 0.024). The distances between samples within groups were smaller than those between groups comparing the wild and captive samples based on PCA (Fig. 6A). Subsequently, the composition of BGCs displayed significant differences among carnivores, herbivores, and omnivores (AMOVA, *P* = 0.040) and between carnivores and herbivores (AMOVA, *P* = 0.018), whereas a similarity was observed between carnivores and omnivores (AMOVA, *P* = 0.133) and herbivores and omnivores (AMOVA, *P* = 0.566). The samples from carnivores and herbivores could be separated in PCA owing to a relatively large distance between them; however, the samples from omnivores could not be distinguished (Fig. 6B). Interestingly, the body size of the mammals mirrored the differences in BGCs (large–middle–small, AMOVA, *P* = 0.023), particularly for large and middle-size animals (AMOVA, *P* = 0.007) (Fig. 6C). In order to further confirm our results, considering that large animals could typically hold large genomic data sets due to their high microbial numbers, we used the ratio of BGCs to the total number of the respective orders to re-conduct the AMOVA test. The *P*-values were 0.029 among large, middle-sized, and small mammals and 0.014 between large and middle-sized mammals. Finally, the threatened class had no significant influence on BGCs (AMOVA, *P* = 0.199) (Fig. 6D). Moreover, the Kruskal–Wallis H test showed that glucorhamnan (BGC0002302) differed significantly among carnivores, herbivores, and omnivores (Fig. S1, *P*<0.05). In addition, polysaccharide (BBGC0001411) differed significantly across the body size (Fig. S1, *P*<0.05). These results suggest that the habitat, diet, and body size play important roles in the metabolic activity of mammalian gut microbiota.

## DISCUSSION

Understanding the biosynthetic potential of microbiomes is one of the most important challenges in understanding the interactions between the gut microbial community and the host. However, studies on the biosynthesis of gut microbes in non-human mammals are limited. Natural products derived from the mammalian gut are now believed to be a class of RiPPs, and a few NRPs and PKs are isolated from soil or aquatic microbes (22, 23). However, previous studies were limited to mining individual genomes (24). TaxiBGC, a taxonomy-guided pipeline for predicting experimentally characterized BGCs based on metagenomic data, suggested that NRPs and PKs were the dominant natural products derived from mammalian gut microbiota, which is inconsistent with the findings of previous studies. Ribosomally synthesized and post-translationally modified peptides are a variety of natural products of ribosomal origin that primarily consist of lantibiotics, bacteriocins, microcins, and thiazole/oxazole-modified microcins (TOMMs) (25). PKs and NRPs are polymerized by related enzymes such as polyketide synthetase and non-ribosomal peptide synthetases (26). This result indicates that the biologically active microbial natural products in the mammalian gut are NRPs and PKs, rather than RiPPs, which are predominant.

One of the contributions of this study is the construction of extensive BGC data sets derived from 53 species and 373 metagenomes. This data set can be used to investigate the distribution of BGCs across mammals and to elucidate the possible mechanisms underlying host microbe symbiosis. We found that carnivorous and omnivorous mammals (Carnivora and Primates) had the most BGCs, followed by large and medium-sized mammals (Artiodactyla and Perissodactyla). The gut microbes of small mammals (Lagomorpha and Rodentia) had the fewest biosynthetic genes. However, this study revealed fewer BGCs in Proboscideans than in Lagomorpha, which may be because only metagenomic data of Asian elephants are available in public databases. The complexity of the food affects the biological functions of gut microbes. Diet drives changes in the abundance and composition of the microbial community (27). Carnivores and herbivores differ in the metabolic profiles encoded by their microbiome, with the microbiome of carnivorous mammals dedicated to protein degradation, whereas that of herbivores is rich in genes associated with the synthesis of essential amino acids (28). The gut microbes of carnivorous mammals have a higher abundance of genes associated with cobalamin and thiamine synthesis than those of herbivores and omnivores (29). Feeding frequency is a core component of carnivorous feeding ecology and can be determined by prey availability, intestinal size, and other ecological factors. Most importantly, eating frequency is negatively correlated with body weight, resulting in large carnivores often having longer intervals between meals (30). For example, the polar bear is the largest land carnivore and eats on average once every 29 days, with long intervals between meals. Another large carnivore, the lion, usually feeds once a week, but can last up to 16 days without eating in the wild (30, 31). In our analysis, mammals with relatively large body masses had a high abundance of genes related to the biosynthesis of metabolites. This may help provide additional energy for survival. Mammalian BGCs can be represented by gene clusters related to the production of yersiniabactin, Pf-5 pyoverdine, colanic acid, and glucurhamnan, which are highly abundant and shared by seven mammalian orders. As previously reported, yersiniabactin helps gut microbes acquire zinc from their hosts, which is an important cofactor for bacterial metabolism (32). The primary function of Pf-5 pyoverdine is the accumulation, mobilization, and transport of iron, which is necessary for cell metabolism (33). Colanic acid produced by gut microbes regulates mitochondrial dynamics and the unfolded protein response (UPRmt) in the host (34). Glucorhamnan is produced by the gut microbe *Ruminococcus gnavus* and is associated with inflammatory diseases in human (35). These results may provide preliminary insights into the common function of natural products that produce mammalian gut microbes that can help maintain gut homeostasis in cell-to-cell communication and play a role in host resistance to inflammation.

The effect of host phylogeny on the composition of BGCs in mammals was further explored. We did not find support for the hypothesis that every mammalian order has unique BGCs, which can only be found in five orders: Carnivora, Lagomorpha, Perissodactyla, Primates, and Rodentia. Small herbivorous mammals have fewer BGCs. The abundance of natural products unique to Rodentia is extremely low. Biosynthetic gene clusters from Lagomorpha are unique in producing geosmin and pyrrolnitrin, which are classified as terpene types. Geosmin can act as a signal (attractant or repellent) or a protective metabolite against biological and abiotic stresses (36). Pyrrolnitrin, a microbial pyrrole salt metabolite with antibacterial properties, is used in the pharmaceutical industry (37). However, the composition of BGCs in large herbivorous mammals differs from that in small herbivorous mammals. The BGC products of kocurin, lagmysin, tacrolimus, and cacibiocin B belonging to NRP, RiPP, and others were only found in Perissodactyla. Kocurin is a thiazolyl peptide that is active against methicillin-resistant *Staphylococcus aureus* (38). Lagmysin is an antibiotic that exhibits varying degrees of cytotoxicity activity (39). Tacrolimus is part of an immunosuppressive regimen (40). Cacibiocin B is a recently discovered aminocoumarin and potent antibiotic (41). The gut microbes of Primates (large omnivorous mammals) can specifically synthesize five natural products: colicin V, N-acyloxyacyl glutamine, tryglysin B, thiocillin I, and lacticin Q. They are attached to lanthipeptides, NRP, RiPPs, and thiopeptides. Colistin, which is produced by gut microbes and classified as a bacteriocin, can improve the adaptability of the host to disease resistance (42). The amino acid glutamine is critically involved in mental and metabolic diseases (43). Tryglysine and thiocillin are antimicrobial peptides that inhibit the growth of opportunistic pathogens (44, 45). Lacticin is a bacteriocin that generally inhibits the growth of Gram-positive bacteria, including *Listeria monocytogenes* and antimicrobial-resistant bacteria (46). Furthermore, diverse biosynthetic genes were identified in large carnivorous mammals. The gut microbes of Carnivora encode 12 BGCs, namely, colicin V, microcin C7, lacticin Q, microcin J25, clostridiolysin S, thiocillin I, gallidermin, clostridiolysin S, carnocyclin, capsular polysaccharides, aranazole, and aerobactin. Among these, colicin V, lacticin Q, and thiocillin I were the same as BGCs in Primates, which might be related to the similarities in diet. Microcin is a viable immunomodulatory peptide that can be used against pathogenic microbial infections (47). Clostridiolysin is a post-translationally modified hemolytic toxin (11). Gallidermin is a lanthionine-containing peptide antibiotic with activity against pathogenic bacteria (48). Carnocyclin is a bacteriocin produced by *Carnobacterium maltaromaticum* UAL307 against numerous Gram-positive bacteria (49). The polysaccharide capsule is the dominant surface structure in an organism and plays a critical role in virulence (50). Aerobactin appears to be an important contributor to extracellular pathogenesis and extracellular stages of intracellular pathogen growth (51). Four large mammals with different diets (Proboscidea, Carnivora, Artiodactyla, and Primates) have certain common BGCs that can biosynthesize biabactin, Pf-5 pyoverdine, glucorhamnan, colanic acid, polysaccharide, meilingmycin, polysaccharide, APE Ec, MA026, Sch-47554, Sch-47555, polysaccharide C, phosphonoglycans, and deoxyhangtaimycin. Some of them are the same as the BGCs in Carnivora and Primates. Meanwhile, MA026 is a natural antiviral compound against the hepatitis C virus (52). Sch-47554 and Sch-47555 are antifungal antibiotics produced by *Streptomyces* (53). Deoxyhangtaimycin shows activity against the influenza A virus, highlighting its potential as an antiviral compound (54). Therefore, we provide evidence that the evolutionary relationship of the host does not have a decisive effect on the biosynthetic capacity of mammalian gut microbes. We suspected that dietary habits and body size may influence BGC synthesis.

Symbiotic gut microbes play an important role in physiology and immune regulation of the host (55, 56). Phylogeny, habitat, diet, and threatened classes are believed to be the major factors shaping the taxonomic membership and functional profiles of gut microbial communities (57–60). These parameters (along with body size) were selected to further screen potential factors influencing the composition of BGCs across mammals. Our results suggest that diet has a significant effect on the composition of BGCs, particularly in carnivores and herbivores. Significant differences in the composition of BGCs were observed between wild and captive groups. We found that diet significantly influences the microbial biosynthesis of glucorhamnan, which is related to the inflammatory diseases (35). This result is rational because research has shown that diet can affect the risk of certain diseases and the effectiveness of treatment (61), and the diet component could shape the structure of the microbial community (62). However, we did not observe any differences in the genes associated with microbial biosynthesis among the threatened classes. Interestingly, we identified certain significant effects of body size on BGCs collected from the gut microbiome of mammals, particularly between large and small-sized mammals. In particular, body size significantly alters the production of polysaccharides in mammals, which plays a key role in virulence (50). As usual, body size has not been considered an influencing factor for the gut microbial community composition and structure. Only one study indicated that body size is a crucial factor in animal metabolism aimed at promoting survival (63) and that small mammals require a lot of energy to maintain a high mass metabolic rate. In this study, four wild flying squirrels with body size scaling were compared, and *Bacteroidetes* was found to account for up to 19% of the small animals, but was absent in large animals, and that small animals had more genes related to carbohydrate and amino acid metabolism than large animals. Actually, the evolution of body size is a fundamental theme in evolutionary biology because it is closely linked to multiple life histories and ecological characteristics (64). Hence, investigating this aspect that links body size scaling to the gut microbial function in mammalian hosts would be interesting. However, this study is limited, in that multiple contributing factors cannot be effectively distinguished. In the future, careful selection of samples will be needed to explore the effects of body size on the gut microbiome, such as the selection of mammals with different body sizes for exploring the effects of the same diet.

In conclusion, our unbiased identification of experimentally characterized BGCs and their annotated products from large-scale metagenomes elucidates potential metabolite-driven interactions between complex microbial communities and their hosts. We identified 2,479 BGCs classified into 25 biosynthetic classes, which enlarged the profiling of the microbial metabolic products in the gut of mammals. The 2,479 distinct BGCs identified here can also help guide natural product discovery and utilize biosynthetic gene information in chemistry. Moreover, we found that variation in BGCs was affected by body size. Body size is a fundamental trait of living organisms that varies greatly between taxa and has important implications for life history and ecology (65–67). A widely accepted pattern, named Cope’s rule, is an active selection toward an increased body size throughout evolution (68). However, current studies have focused on the plasticity of gut microbes caused by environmental factors. A long-ignored research interest is the role of host genetics in shaping gut microbial populations. We proposed that body size might drive the variation in gut microbial metabolism, which will aid in future investigations of the mammal gut microbiome. The BGCs constructed from metagenomic data considerably improve our understanding of the biochemical functions of mammalian microbiota and shed light on microbial adaptations to the mammalian gut.

## MATERIALS AND METHODS

### Metagenomic data collection

Fecal samples of Felidae were collected between April and June 2023 from Beijing Wildlife Zoo, Beijing Zoo, the Breeding Center of Beijing Zoo, Chongqing Zoo, and Xining Wildlife Zoo. All fecal samples were immediately frozen in dry ice after collection and transported to the laboratory for storage at −80°C until further processing. Published animal gut metagenome reads were downloaded from the National Genomics Data Center (https://ngdc.cncb.ac.cn) and National Center for Biotechnology Information (https://www.ncbi.nlm.nih.gov). Detailed information on sample collection and metagenomic data can be found in the Tables S3 and S4.

### Bioinformatic analysis and BGC prediction

The E.Z.N.A. Soil DNA Kit (Omega Bio-tek, U.S.) was used to extract total DNA from the collected feces, according to the manufacturer’s protocol. TBS-380, NanoDrop 2000, and 1% agarose gels were used to assess DNA concentration, purity, and integrity, respectively. The DNA extract was fragmented to an average size of approximately 400 bp using Covaris M220 (Gene Company Limited, China) for paired-end library construction. NEXTFLEX Rapid DNA-Seq (BioScientific, U.S.) was used to construct a DNA library. Adapters containing the full complement of sequencing primer hybridization sites were ligated to the blunt end of fragments. Qualified DNA libraries following PCR amplification were sequenced on the Illumina NovaSeq platform (Illumina Inc., San Diego, CA, USA). Trimmomatic and Fast QC were used for quality control for all metagenomic sequences. Bowtie2 was applied to remove the host information. TaxiBGC (https://github.com/danielchang2002/TaxiBGC_2022) was used to predict BGCs and products. The TaxiBGC pipeline consists of three steps: (1) metagenomes were analyzed at species level using MetaPhlAn3 (2). The first prediction of BGC was performed by querying these species (identified in step 1) in the TaxiBGC reference database, and 390 unique species with their experimentally characterized BGCs and known SMs were collected (3); the predicted BGCs (from the second step) were confirmed based on the read mapping (i.e., alignment) using BBMap (21). TaxiBGC has been applied on 2,650 metagenomes, showing that this pipeline can satisfactorily predict BGCs (mean F1 score, 0.56; mean PPV score, 0.80) (21).

### Mathematical analysis

Non-redundant BGCs were retained in the species with multiple samples. The value of a BGC in an order is the average of the BGCs across all species in that order. Principal component analysis, UpsetVenn, AMOVA and ANOSIM analyses, Wilcoxon rank-sum test and Kruskal–Wallis H test were performed using R 4.3.0.

## Data Availability

The raw sequence data reported in this paper have been deposited into the Genome Sequence Archive (Genomics, Proteomics, & Bioinformatics 2021) in the National Genomics Data Center (Nucleic Acids Res 2022), China National Center for Bioinformation / Beijing Institute of Genomics, Chinese Academy of Sciences, under the accession number of CRA014688.

## SUPPLEMENTAL MATERIAL

Figure S1 spectrum.00864-24-s0001.pdf The changed BGCs between captive and wild.

Table S1 spectrum.00864-24-s0002.xlsx The generated mammalian gut metagenome covering seven orders.

Table S2 spectrum.00864-24-s0003.xlsx The identified 2,479 unique experimentally characterized BGCs.

Table S3 spectrum.00864-24-s0004.xlsx The downloaded metagenomic data.

Table S4 spectrum.00864-24-s0005.xlsx The collected samples from Felidae.

ASM does not own the copyrights to Supplemental Material that may be linked to, or accessed through, an article. The authors have granted ASM a non-exclusive, world-wide license to publish the Supplemental Material files. Please contact the corresponding author directly for reuse.
