# Supplementary material for: Mining metagenomic data to gain a new insight into the gut microbial biosynthetic potential in placental mammals
Source: Microbiol Spectr. 2024 Aug 20;12(10):e00864-24. doi: 10.1128/spectrum.00864-24 (PMC11448209; doi:10.1128/spectrum.00864-24)

Figure S1. The changed BGCs between captive and wild (A); among herbivore, carnivore and omnivore (B); among large, middle and small (C).

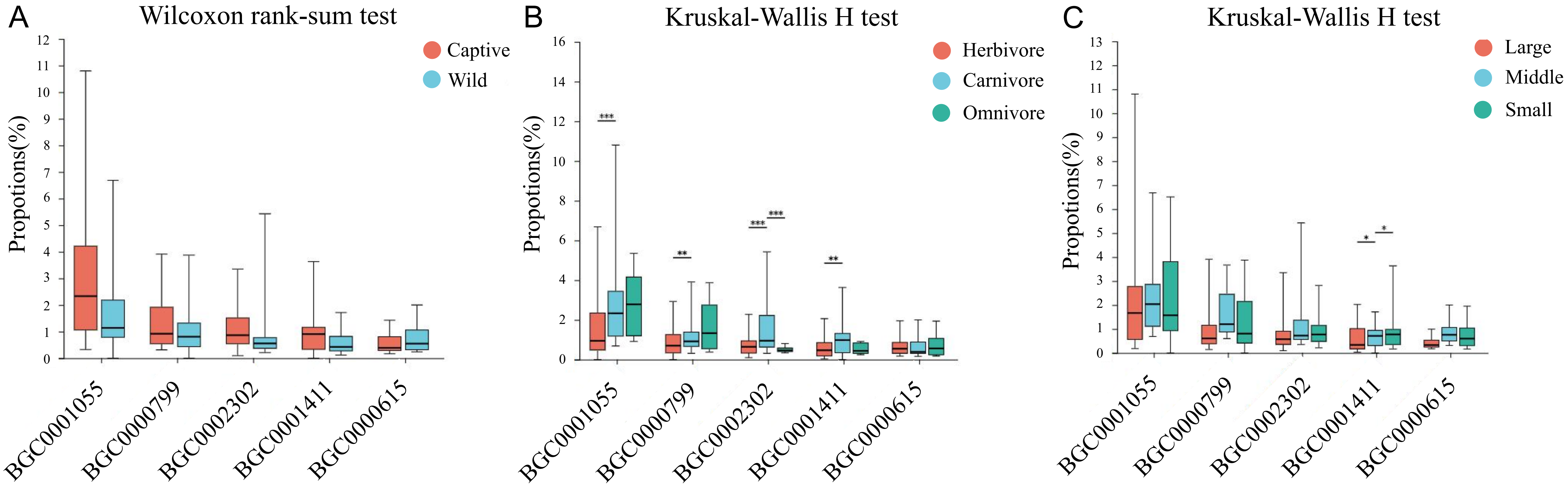

Supplement: Figure S1 — The changed BGCs between captive and wild. [file spectrum.00864-24-s0001.pdf]
